# Fever and Ague

**Published:** 1883-10

**Authors:** 


### FEVER AND AGUE.

FEVER and Ague, Chills and Fever, Malarial Fever and Intermittent Fever, popularly speaking, are the same thing. There is but one cause for such fever—namely, TORPID LIVER; and but one cure, and that is a remedy that will promptly restore the liver to the performance of its proper functions.

Fever and Ague is a disease that cannot be, with safety, neglected; since it thus may become the starting-point of more serious maladies. Tampering with an infinite variety of remedies is a great evil. Almost every man you meet has a *certain cure* for this disease; and by the time a patient has tried a few such remedies he has so complicated and intensified the trouble that he is lucky if he escapes without some disease of the liver that will last a lifetime. The best time to treat an ailment is at its commencement. It is then most frequently a derangement of an important function rather than a disease; and it is easy to set a wheel in motion before it has fairly stopped. A small quantity of medicine that will act on the liver will, taken in time, arrest fever and ague. But there are but two or three remedies that act with sufficient promptness. There is a popular notion that any medicine that affects the bowels is the proper thing for that disease. No one was ever cured by taking salts or castor oil, or pills "*warranted purely vegetable,*" or any such nonsensical thing, but a single dose of LIVER PILLS will cure any recent case of fever and ague; and two or three doses, at proper intervals, will cure any curable case. They cure not because they are pills, but because they are composed of remedies especially adapted to liver troubles. Crushed into a powder they would produce the same effect. Many persons think that *pills* are *pills*, and that one kind is the same as any other. It is about as reasonable to ask a druggist for *some medicine*, without stating *what kind* of medicine.

Great surprise has been manifested at the appearance of fever and ague, within the last few years, in places where it was never known before: among the hills of New England and on the tops of its mountains. The readers of the JOURNAL will remember that we never regarded the appearance of malarial fever in those places as in the least mysterious. We have always contended that the source of the disease is in the water supply, and that a thorough investigation would prove the truth of our assertion in every instance. We know from experience that a family may enjoy the best of health for years in a so-called malarial district, when all the water used for drinking and cooking is pure. Reforms move slowly, but the time will come when when the first care of every intelligent family will be to secure a water supply beyond suspicion of contamination. "An ounce of prevention is worth a pound of cure."

